# RETRACTED: Kshirsagar et al. A Combination Therapy of Urolithin A+EGCG Has Stronger Protective Effects than Single Drug Urolithin A in a Humanized Amyloid Beta Knockin Mice for Late-Onset Alzheimer’s Disease. *Cells* 2022, *11*, 2660

**DOI:** 10.3390/cells15141307

**Published:** 2026-07-22

**Authors:** Sudhir Kshirsagar, Rainier Vladlen Alvir, Jangampalli Adi Pradeepkiran, Ashly Hindle, Murali Vijayan, Bhagavathi Ramasubramaniam, Subodh Kumar, Arubala P. Reddy, P. Hemachandra Reddy

**Affiliations:** 1 Department of Internal Medicine, Texas Tech University Health Sciences Center, 3601 4th Street, Lubbock, TX 79430, USA; sudhir.kshirsagar@ttuhsc.edu (S.K.); raalvir@ttuhsc.edu (R.V.A.); pradeep.jangampalli@ttuhsc.edu (J.A.P.); ashly.hindle@ttuhsc.edu (A.H.); murali.vijayan@ttuhsc.edu (M.V.); b.ramasubramaniam@ttuhsc.edu (B.R.); subodh.kumar@ttuhsc.edu (S.K.); 2 Center of Emphasis in Neuroscience, Department of Molecular and Translational Medicine, Texas Tech University Health Sciences Center, El Paso, TX 79905, USA; 3 Nutritional Sciences Department, College of Human Sciences, Texas Tech University, 1301 Akron Ave, Lubbock, TX 79409, USA; arubala.reddy@ttu.edu; 4 Department of Pharmacology and Neuroscience, Texas Tech University Health Sciences Center, Lubbock, TX 79430, USA; 5 Department of Neurology, Texas Tech University Health Sciences Center, Lubbock, TX 79430, USA; 6 Department of Public Health, Graduate School of Biomedical Sciences, Texas Tech University Health Sciences Center, Lubbock, TX 79430, USA; 7 Department of Speech, Language, and Hearing Sciences, Texas Tech University Health Sciences Center, Lubbock, TX 79430, USA

The journal retracts the article titled “A Combination Therapy of Urolithin A+EGCG Has Stronger Protective Effects than Single Drug Urolithin A in a Humanized Amyloid Beta Knockin Mice for Late-Onset Alzheimer’s Disease” [1], cited above.

Following publication, concerns were brought to the attention of the publisher concerning the integrity of several figures presented in this article [1].

According to the journal’s standard procedures, the Editorial Office and Editorial Board conducted an investigation that identified indications of inappropriate image editing and duplication in Figures 4, 6, 12 and 13, including the reuse of an image from an earlier publication by the same authors. Additionally, the investigation identified concerning citation patterns present within this publication. Although the authors collaborated during the investigation and provided supporting materials, the Editorial Board concluded that the concerns could not be resolved satisfactorily based on the available information.

Consequently, the Editorial Board has lost confidence in the reliability of the reported findings and has decided to retract this publication, as per MDPI’s retraction policy. 

This retraction was approved by the Editor-in-Chief of the journal *Cells*. 

The authors have been informed of this retraction and have indicated their disagreement with this decision.
